# Exploring Viral Diversity in a Unique South African Soil Habitat

**DOI:** 10.1038/s41598-017-18461-0

**Published:** 2018-01-08

**Authors:** Jane Segobola, Evelien Adriaenssens, Tsepo Tsekoa, Konanani Rashamuse, Don Cowan

**Affiliations:** 1 Biosciences Unit, Council for Scientific and Industrial Research (CSIR), Pretoria, South Africa; 2 Centre for Microbial Ecology and Genomics, University of Pretoria, Pretoria, South Africa

## Abstract

The Kogelberg Biosphere Reserve in the Cape Floral Kingdom in South Africa is known for its unique plant biodiversity. The potential presence of unique microbial and viral biodiversity associated with this unique plant biodiversity led us to explore the fynbos soil using metaviromic techniques. In this study, metaviromes of a soil community from the Kogelberg Biosphere Reserve has been characterised in detail for the first time. Metaviromic DNA was recovered from soil and sequenced by Next Generation Sequencing. The MetaVir, MG-RAST and VIROME bioinformatics pipelines were used to analyse taxonomic composition, phylogenetic and functional assessments of the sequences. Taxonomic composition revealed members of the order Caudovirales, in particular the family *Siphoviridae*, as prevalent in the soil samples and other compared viromes. Functional analysis and other metaviromes showed a relatively high frequency of phage-related and structural proteins. Phylogenetic analysis of *PolB*, *PolB2*, *terL* and *T7gp17* genes indicated that many viral sequences are closely related to the order Caudovirales, while the remainder were distinct from known isolates. The use of single virome which only includes double stranded DNA viruses limits this study. Novel phage sequences were detected, presenting an opportunity for future studies aimed at targeting novel genetic resources for applied biotechnology.

## Introduction

The Cape Floristic Region situated in the Western Cape province of South Africa is one of five Mediterranean-type ecosystems in the world^1^ and is recognized as one of the world’s biodiversity hotspots^2^. Fynbos (fine bush) is the main vegetation type of this region with the *Proteaceae*, *Ericaceae* and *Restionaceae* families dominating Kogelberg Biosphere Reserve Fynbos vegetation. Within this region, the fynbos comprises approximately 9000 plant species of which 70% are endemic to the region^1,3^. Fynbos vegetation types survive on highly heterogeneous, acidic, sandy, well-leached and infertile soils. The fynbos plants also survive invasions by foreign plants^4^ and seasonal drought conditions^5^.

Microorganisms make up a great proportion of the living population in the biosphere. They provide important ecosystem services in edaphic habitats^6^ and form complex symbiotic relationships with plants^7^. Plant-associated microorganism studies have shown high microbial diversity in fynbos soils^2^, where they play a role in sustaining plant communities^8^. A study focusing on the linkage between fynbos soil microbial diversity and plant diversity showed the presence of novel taxa and of bacteria specifically associated with the rhizospheric zone^9^. Studies on ammonium-oxidizing bacteria demonstrated that plant-species specific and monophyletic ammonium oxidizing bacterial clades were present in fynbos soils^10^, where abundance might be driven by the acidic and oligotrophic nature of these soils^11^. There is evidence that above-ground floral communities are implicated in shaping microbial communities^12,13^, and that some microbial clades show a high level of plant–host specificity^10^. This is consistent with the general concept of the mutualistic relationships between the plants and the microbial communities in fynbos soils^14^.

Soil-borne viruses, including phages, are of great importance in edaphic habitats due to their ability to transfer genes from host to host and as a potential cause of microbial mortality (leading to changes in turnover and concentration of nutrients and gases), processes that can profoundly influence the ecology of soil biological communities^15^. Virus diversity associated with fynbos plants from Kogelberg Biosphere Reserve fynbos soil has never been thoroughly investigated^16^. The difficulty of culturing viruses, which are absolutely dependent on a cell host to provide the apparatus for replication and production of progeny virions, presents a barrier to fully accessing viral biodiversity. This is a particular issue in poorly studied habitats, such as fynbos soil, where the true microbial (host) diversity is largely unknown and most microbial phylotypes have never been cultured^17^. The biodiversity and ecology of viruses in many soils therefore remain poorly investigated and poorly understood^18^.

Metaviromic surveys of terrestrial environments such as hot desert soil^18^, rice paddy soil^19,20^, Antarctic cold desert soil^21,22^ and hot desert hypolithic niche communities^23^ have been reported in recent years and have significantly advanced the field of soil viral ecology^20,24^. These studies have also facilitated the discovery of novel virus genomes^20,22,23^ and novel viral enzymes^25^.

However, surveys of viral diversity using NGS sequencing techniques in conjunction with metaviromic databases have focused principally on aquatic environments^26–28^. Studies on taxonomic composition using public metaviromic databases for viral diversity estimations have shown that a majority of environmental virus sequences are unknown^19^: ~70% of sequences have no homologs in public databases and are therefore typically labelled “viral dark matter”^29,30^. Bacteriophages constitute the largest known group of viruses found in both aquatic^24,31^ and soil environments^32,33^.

Here we report the first investigation of virus diversity in a unique soil type (fynbos soil) using metaviromic approaches. The metavirome of Kogelberg Biosphere Reserve fynbos soil was characterised in terms of diversity and functional composition and adds a new level of understanding to the exceptional biodiversity of this habitat.

## Results and Discussion

### Viral Morphology

Analysis of the morphology of viruses identified in Kogelberg Biosphere Reserve fynbos soil was carried out by transmission electron microscopy (TEM). TEM analysis of the virus preparations showed that the majority of the isolated virus particles were morphologically similar to known virus taxonomic groups^34^. The isolated virus particles from the fynbos soil were tailed, spherical or filamentous (Supplementary Fig. S1). Various particles with head-tail morphology, typically belonging to the families *Myoviridae*, *Siphoviridae* or *Podoviridae*, were observed.

These results are in a good agreement with previously published findings showing the high dominance of tailed phages in soils from various geographic areas^24,33,35^. The undetermined spherical or filamentous morphologies in TEM micrographs could be *bona fide* but uncharacterised viral structures. Spherical particles resembling capsid structures could be members of the *Leviviridae*, *Partitiviridae*, *Chrysoviridae*, *Totiviridae* or *Tectiviridae* families, or small plant viruses^34^. Filamentous particles may possibly correspond to the virus structures of the *Inovirus* genus, the members of which contain circular ssDNA within flexible filamentous virions. The presence of spherical types and filamentous type of virus particles was also reported for Delaware soils^32^. The aggressive extraction procedure used in the current study may have resulted in a high incidence of phage tail breakage and the generation of tailless phages^36^.

### Metavirome Assembly

Assembly of the DNA sequence reads yielded 13,595 contigs larger than 500 bp, with an average length of 2,098 bp, accounting for a total of 28,526,478 bp (Table 1). Two different metagenomics pipelines; MetaVir^37^ and VIROME^38^, were used for analysis of the contigs, while MG-RAST^39^ was used for the analysis of the uploaded reads (Table 2). The MetaVir pipeline predicted 51,274 genes, with 5,338 affiliated contigs (i.e., contigs with at least one BLAST hit) and 7880 unaffiliated contigs (Table 2). MetaVir compares reads/contigs to complete viral genomes from the Refseq database and is specifically designed for the analysis of environmental viral communities^37^. The VIROME pipeline^38^ predicted 51,242 protein coding regions. Of these, 9555 were assigned as functional proteins, and 31,109 were unassigned (Table 2). Comparisons of functional and taxonomic analysis between Virome and MetaVir indicate that many of the predicted genes were overlapping between the two pipelines with MetaVir on average having a higher predictive potential (Supplementary Table S1). The MG-RAST pipeline predicted 2,555,524 protein coding regions. Of these predicted protein features, 119,220 were assigned a functional annotation using protein databases (M5NR)^40^ and 2,362,076 had no significant similarities to sequences in the protein databases (ORFans). MG-RAST core analysis and annotation depends heavily on the SEED database which is largely comprised of bacterial and archaeal genomes^41^. The majority of the annotated sequences in MG-RAST were mapped to bacterial genomes. This high percentage of bacterial sequences in metaviromes may be due to the presence of unknown prophages in bacterial genomes, phages carrying host genes, relatively large size of bacterial genomes compared to viral genomes and larger size of the microbial genome database which is statistically increasing the chance of matching bacterial sequences. The MG-RAST pipeline was used to analyse the reads, not the contigs and shows, therefore a higher number of predicted features, including more partial CDSs^42^ No rDNA sequences were found with the MG-RAST and VIROME pipelines, confirming the viral origins of the DNA. The fact that more than 80% of the hits in this study, consistent with previous viral metagenomics studies^31,43,44^, were assigned as hypothetical proteins derived from unknown viruses suggests the presence of a substantial pool of novel viruses.

**Table 1:** Next Generation sequencing data analysis. Representation of the assembly, annotation, and diversity statistics produced by CLC Genomics

Features	CLC
#Pre-QC Sequence reads	7,019,527
#Pre-QC sequence in base pairs	1,488,462,918
#post-QC average read length	212.05
#contigs	13,595
#contigs/reads in bp	28,526,478 bp

**Table 2:** Comparison of the automated pipelines; such as MetaVir (contigs), VIROME (contigs) and MG-RAST (reads), used to characterize the Kogelberg Biosphere Reserve. *Affiliated CDS are CDS with homologues in at least one of the databases used, while ORFans are predicted ORFs which have no database homologue.

Features	MetaVir	MG-RAST	VIROME
#predicted CDS	51,274	2,555,524	51,242
#affiliated CDS*	5,868	119,220	9,555
#ORFans*	45,406	2,362,076	31,109
#rRNAs	NA	0	0
Database used for CDS annotation	RefSeq virus, pfam	GenBank, IMG, KEGG, PATRIC, RefSeq, SEED, SwissProt, TrEMBL, eggNOG, COG, NOG, KOG,	KEGG, SEED, COG, GO, UniRef100, PHGSEED, MgOI, ACLAME

### Viral Diversity Estimation And Taxonomic Composition

**Figure 1:**
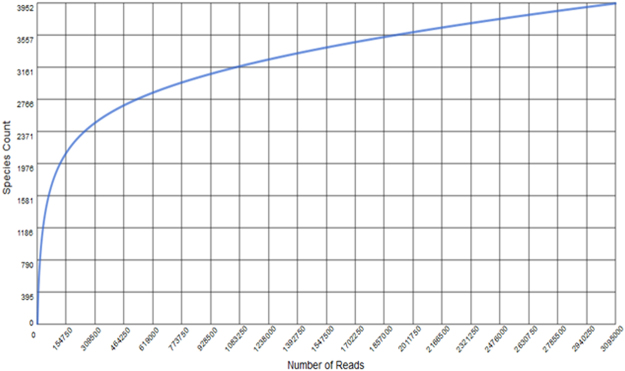
Rarefaction curve of the Kogelberg Biosphere Reserve fynbos soil metavirome. Clustering was set at 90% similarity.

The rarefaction curve computed by MG-RAST showed 3952 species clusters at 90% sequence identity for the 3,095,000 reads. The curve did not reach an asymptote (Fig. 1), although extrapolation suggested that approximately 78% of the viral diversity was covered by the metavirome sequence dataset.

MetaVir was used for viral taxonomic composition analysis of the contigs. The taxonomic composition was computed from a BLASTp comparison of the predicted proteins in the contigs with the Viral Refseq protein database (release of 2016-01-19). The results revealed that 37.6% of the contigs represented a significant hit (threshold of 50 on the BLAST bit score). MetaVir identified 18 virus families, in which prokaryotic viruses were the most abundant and dominated by the order *Caudovirales*, consistent with the TEM observations. The relative abundance ranking of the different families was as follows: tailed bacteriophage families *Siphoviridae* > *Myoviridae* > *Podoviridae*, followed by the algae-infecting family *Phycodnaviridae*, the archaeal virus family *Ampullaviridae* and the amoeba-infecting family *Mimiviridae* (Table 3). Surprisingly, large viruses belonging to the families *Phycodnaviridae* and *Mimiviridae* were detected, which should have been removed during the filtration process due to the use of a 0.22-µm filtration step to remove bacterial cells. The identification of *Mimiviridae* suggests that this filtration process allowed partial mimivirus particles or free-floating DNA to pass through the membrane. Mimiviruses appear to infect only species of *Acanthamoeba*, which are ubiquitous in nature and have been isolated from diverse environments including freshwater lakes, river waters, salt water lakes, sea waters, soils and the atmosphere^35,45–47^. This suggests the existence of Mimivirus relatives in the KBR soil.

**Table 3:** Taxonomic abundance. Representation of taxonomic abundance of identified viral ORFs BLASTp with threshold of E value10^−5^ identified by MetaVir.

Virus Order and family	Hosts	Relative abundance of taxa
*Caudovirales*		
***Myoviridae***	Bacteria, Archaea	29
***Podoviridae***	Bacteria	23
***Siphoviridae***	Bacteria, Archaea	45
*Herpesvirales*		
***Herpeviridae***	Vertebrates	0.04
Virus Family and groups not assigned in to Order		
***Phycodnaviridae***	Algae	2
***Ampullaviridae***	Archaea	0.9
***Mimiviridae***	Amoebae	0.8
***Salterprovirus***	Archaea	0.7
***Tectiviridae***	Bacteria, Archaea	0.5
***Iridoviridae***	Vertebrates (Amphibians, Fishes), Invertebrates	0.1
***Marseilleviridae***	Amoeba	0.04
***Nudiviridae***	Arthropods	0.04
***Poxviridae***	Human, Arthropods, Vertebrates	0.02
***Baculoviridae***	Invertebrates	0.02
***Bicaudaviridae***	Archaea	0.02
***Turriviridae***	Archaea	0.02
***Asfarviridae***	Swine	0.02
***Retroviridae***	Vertebrates	0.02
Virus not assigned into Family		
**Unclassified dsDNA phages**	Bacteria	2
**Unclassified dsDNA virus**	NA	4
**Unclassified ssDNA Viruses**	NA	0.07
**Unclassified phages**	Bacteria	2

Other viral families and unclassified viruses (dsDNA and ssDNA) were found in low numbers. Putative contamination of *Enterobacteria* phage phiX174 was also detected in our metavirome sequences. This phage is used for quality control in sample preparation for high-throughput sequencing. Seven sequences from this dataset are similar to the phiX174 genome and were thus disregarded in the taxonomic composition as an artefact of sample processing. Plant viruses were not identified in the dataset, most probably because the majority of plant viruses are RNA viruses which were not sampled in this study.

**Figure 2:**
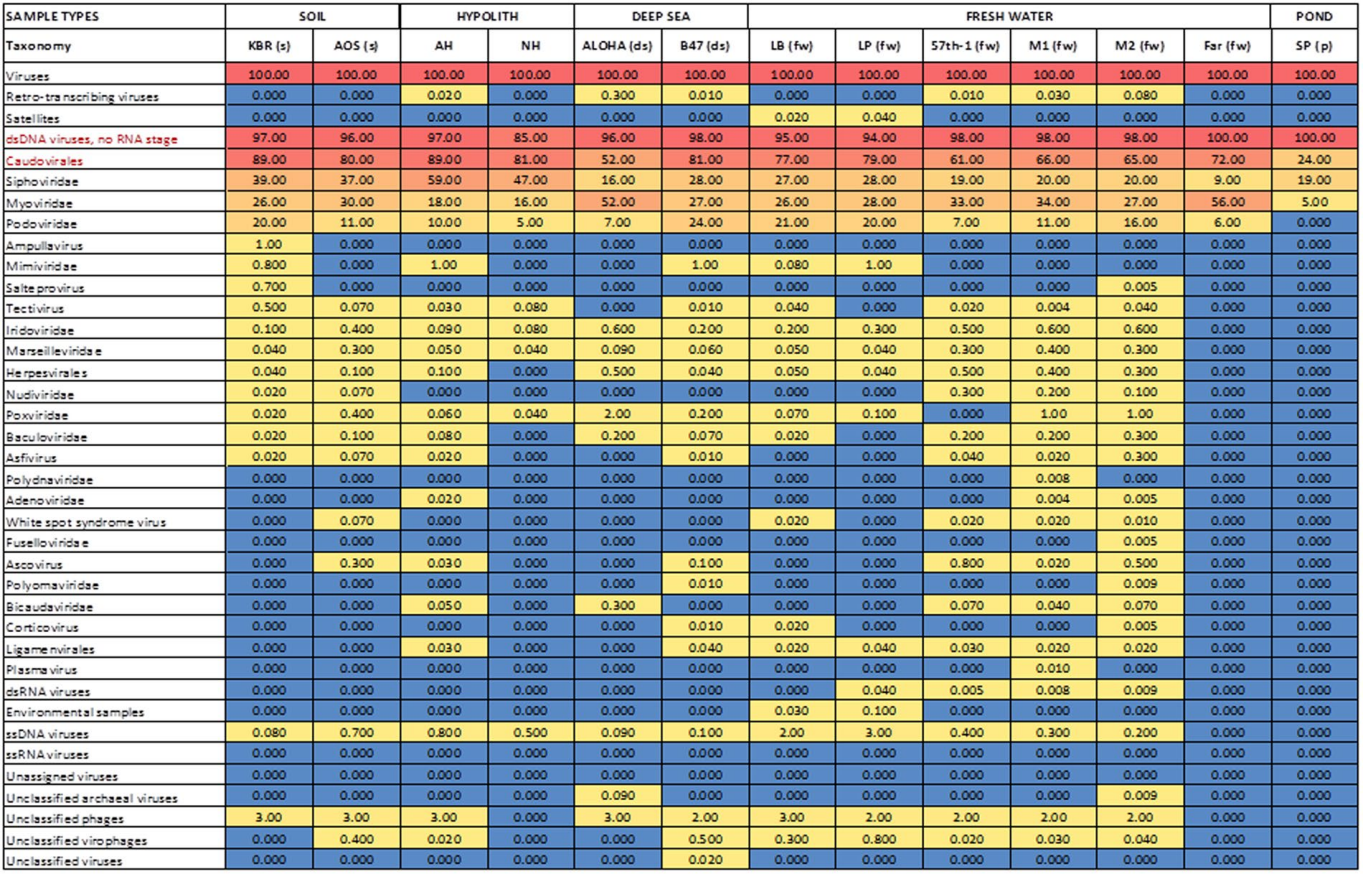
Comparison of the Kogelberg Biosphere Reserve metavirome taxonomic composition with selected publically available metaviromes. Abundances normalized according to predicted genome size with the GAAS tool. Blue colour represents 0.000 taxon, yellow represents 0.01–19.00, mustard represents 20.00–29.00, light red represents 30.00–49.00, and red represents 50.00–100.00 taxon. More details on the description of metaviromes are described in Supplementary Table S3.

The viral composition of Kogelberg Biosphere Reserve fynbos soil was compared to 12 previously published metaviromes from both similar and dissimilar environments, including fresh water^28^, soil and hypolithic niche communities^22,23^, pond water^27^ and sea water^48^ (Fig. 2). A comparative metaviromics approach was used to investigate the assumption that certain environments will select for specific viruses^49,50^.

The *Caudovirales* taxon dominated all metaviromes. In particular, members of the family *Siphoviridae* were dominant in most metaviromes except for some of the freshwater samples, in which myoviruses were dominant. Within the dsDNA viruses, members of rare taxonomic groupings such as the genera *Tectivirus*, *Asfivirus* and *Salterprovirus*, the families *Mimiviridae*, *Iridoviridae*, *Marselleviridae*, *Nudiviridae*, *Poxviridae* and *Baculoviridae* and the order *Herpesvirales* were detected in soil samples as well as in hypolith, deep sea, and freshwater metaviromes. Archaeal virus signatures belonging to the family *Ampullaviridae* have been observed only in the Kogelberg Biosphere Reserve fynbos soil. This family contains viruses with pleomorphic morphologies and a dsDNA genome, and the type species infects the thermoacidophile *Acidianus convivator*, isolated from Italian hot springs^51^. Fresh Water Lake, Antarctic soil and coral metaviromes showed a high abundance of ssDNA viruses, results possibly biased by the use of phi29 polymerase amplification (MDA) of the metaviromic DNA during library construction. The amplification of metaviromic DNA using phi29 polymerase amplification (Multiple Displacement Amplification) has been reported to be biased towards ssDNA templates^19^. It is notable, however, that a high abundance of ssDNA viruses has been observed in beach freshwater samples^52^, where amplification was not used in the preparation of metagenomic DNA. However, in general, other metaviromes which were not amplified using MDA showed a very low number of ssDNA viruses. In general, soils or soil-associated habitats seem to harbour relatively fewer ssDNA viruses and more tailed phages than aquatic ecosystems.

Consistent with other data^22,24,43^, it was found that bacteriophage sequences in Kogelberg Biosphere Reserve fynbos soil made up the majority of the virus fraction. Bacteriophages are common in the environment and are the dominant viral type recovered from metaviromics analyses in soil environments^18,20,23,30^. This finding was not surprising, given the observations from previous studies^35,53^ which showed high prokaryotic abundances in the Kogelberg soil environment. Nevertheless, signature sequences from large dsDNA eukaryotic virus families such as *Mimiviridae* ^54^ were represented in the Kogelberg Biosphere Reserve library despite the use of small pore size filters in sample preparation. Mimivirus signatures have been reported previously in other soil habitats^22^. Sequences that were found to be most similar to mimivirus ORFs were also obtained from Sargasso sea water samples, suggesting that these viruses, and their hosts, have a rather cosmopolitan distribution^46^.

### Phylogeny Of The Kogelberg Biosphere Reserve Fynbos Soil Metavirome

Specific markers targeting virus families or species were used to analyse the taxonomic affiliations of the annotated ORFs and analyse the diversity within the group (reviewed in ref.^55^). Phylogenetic trees were drawn from metavirome sequences on the basis of homology to marker gene reference sequences from the PFAM database. Sequences homologous to the marker genes (*polB*, *polB2*, *T7gp17* and *terL* (Supplementary Fig. S2, S3, S4 and S5) and reference sequences were used to draw phylogenetic trees.

Using the DNA polymerase family B (polB) marker gene, conserved in all dsDNA viruses, Kogelberg Biosphere Reserve sequences appeared to be distantly related to *Rhodothermus* phage RM378 (order *Caudovirales*, family *Myoviridae*). This phage is the only sequenced representative of the “Far T4” group of myoviruses (i.e., distantly related to *Escherichia virus T4*) found in a previous diversity analysis of sequences from French lakes^28^. The Kogelberg polB sequences from this study as well as the gp23 and gp20 marker gene sequences from the French lake study contribute to the expansion of the “Far T4”-like phages dataset.

A DNA polymerase family B (*polB2*) marker gene, which is conserved in members of *Adenoviridae*, *Salterprovirus*, and *Ampullaviridae* and *Podoviridae* family viral groups, was analysed. The analysis showed a separate clade of sequences from the Kogelberg Biospheres reserve soil samples. Other *polB2* sequences from our dataset were found to be distantly related to members of the *Adenoviridae* family (isolated from a wide range of animal sources), the *Podoviridae* family (such as *Mycoplasma* phage *P1*, *Clostridium* phage *phi24R*, *Bacillus* phages *B103*, *phi39*, *Ga1*), the *Ampullaviridae* family (such as *Acidianus-*bottle-shaped virus) and the *Tectiviridae* family (such as *Bacillus* phages *G1L16C*, *Bam35C and AP50*).

Analysis of the metavirome sequence database using the marker gene *T7gp17* showed the presence of members of the *Podoviridae* family, subfamily *Autographivirinae* and genus *Phikmvvirus* and *T7virus*. Members of the genus *phikmvvirus* such as *Pseudomonas* phage LKA1, and unclassified phiKMV phages such as *Ralstonia* phage RSB1, were found to be closely related to the Kogelberg Biosphere Reserve sequences. Currently unclassified members of the genus *T7virus*, such as *Klebsiella* phage K11 and *Yersinia* phage φYeO3–12, were also found to be closely related to sequences in the Kogelberg Biosphere Reserve metavirome. The phages in the subfamily *Autographivirinae* are known to infect a wide range of environmentally important bacteria^56^.

**Figure 3:**
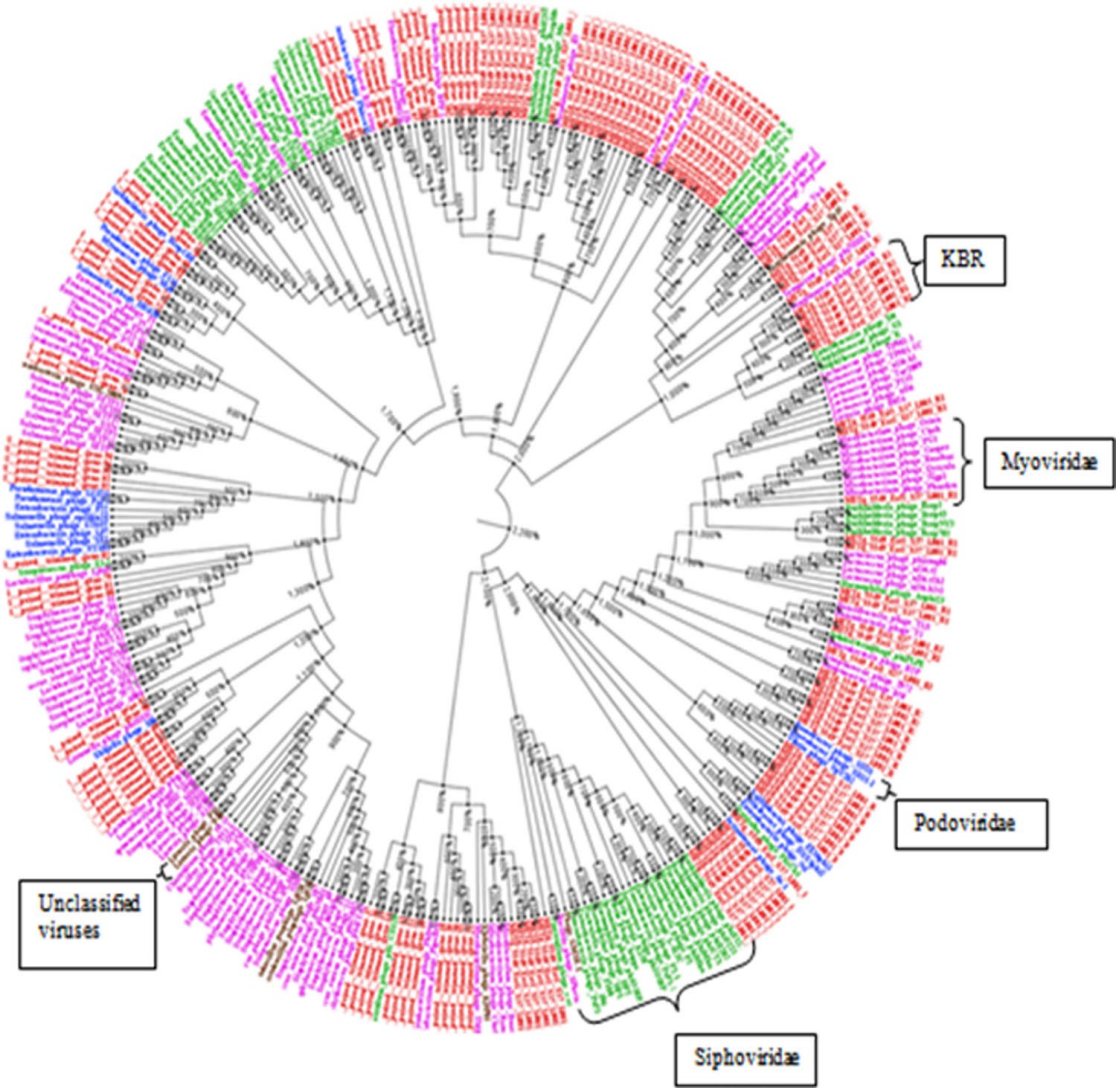
terL phylogenetic tree. Viral sequence origin of Caudovirales indicated with different colours on the contigs names. Kogelberg Biosphere Reserve fynbos soil - Red, Siphoviridae - green, Myoviridae - purple, Podoviridae - blue, unclassified viruses - grey.

Tailed phages of the order *Caudovirales* were the most commonly observed DNA viruses in the Kogelberg Biosphere Reserve sequences, consistent with other environmental samples^23,33,57^. A phylogenetic tree built from a *Caudovirales*-specific terminase large subunit marker gene (*terL*) was used to visualise the diversity of the Kogelberg Biosphere Reserve fynbos soil *Caudovirales* (Fig. 3). The Kogelberg Biosphere Reserve sequences clustered with all three families of tailed phages, indicating high phage richness in our sample set. These results were consistent with the taxonomic affiliations of contigs in the virus families shown in Table 3.

### Analysis of A Near-Complete Phage Genome

**Figure 4:**
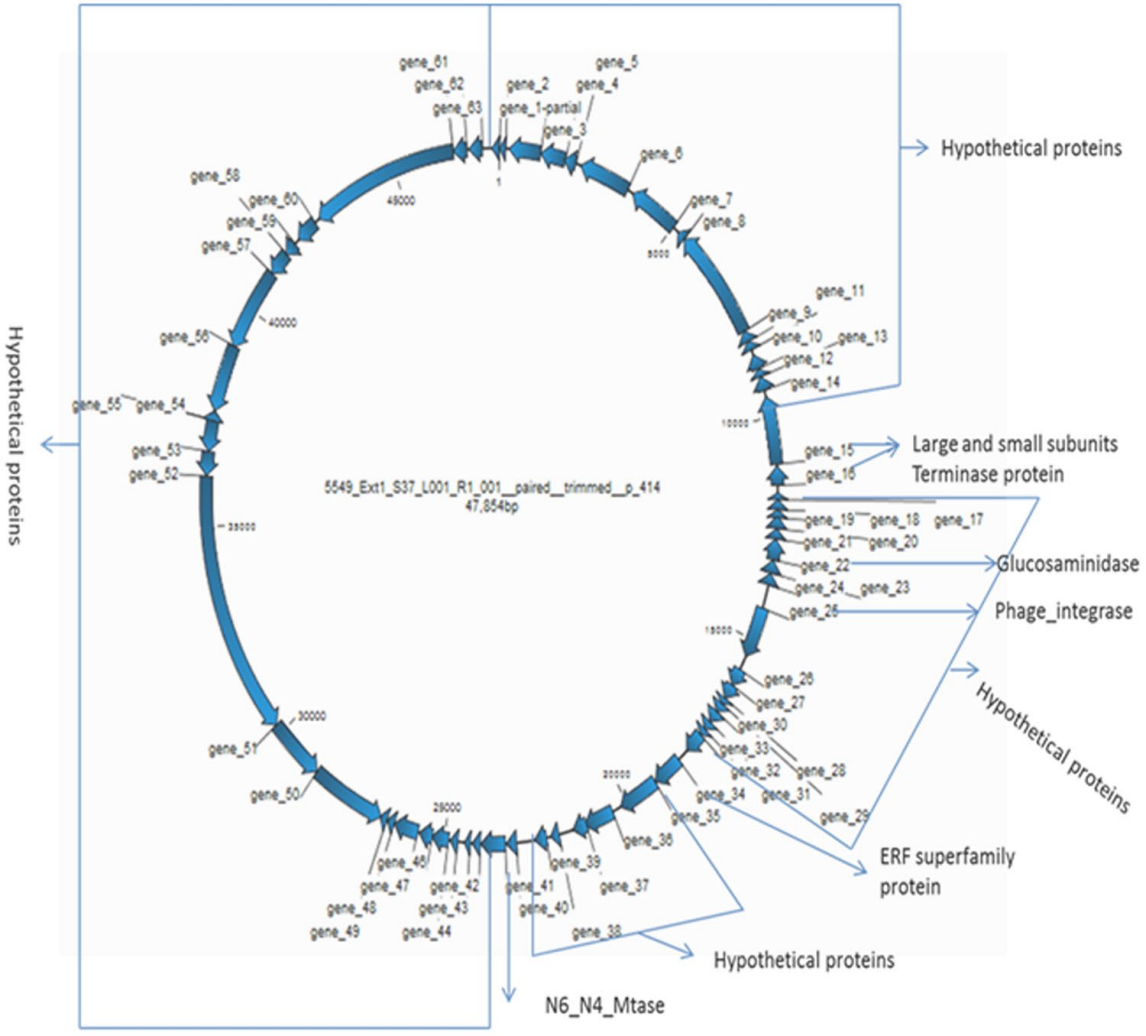
Gene annotation of contig 414. Arrowed blocks are open reading frames (ORFs), showing their orientation. Numbers within the contiguous genome are nucleotide positions, starting within gene number 1 and onwards in a clockwise orientation.

MetaVir assemblies predicted 352 genes from the 6 contigs larger than 40 kb, as well as 758 genes predicted from 19 contigs of between 20 kb and 40 kb. The 6 largest contigs were predicted to be linear, double stranded genomes. The sizes of the genomes were predicted to be 47 kb long with 63 genes for the largest contig (Fig. 4), followed by 44 kb with 58 genes, 42 kb with 61 genes, 42 kb with 53genes, 40 kb with 68 genes and 40 kb with 49 genes. The genes in these contigs were predicted to show similarity to members of the order *Caudovirales*.

The largest contig represents a near-complete phage genome in the family *Podoviridae*. Members of this family typically contain double stranded and linear genomes of around 40–45 kb in length with approximately 55 genes^58^. Four of the genes in this assembled genome (genes 15, 16, 34 and 41) showed similarity to members of both *Podoviridae* and *Siphoviridae* families. The translated products of two of these genes (15 and 16) were identified as putative terminase large subunit (gene 15) and terminase small subunit (gene 16) genes, with 88% and 89% amino acid identity to *Puniceispirillum* phage HMO-2011 and *Pseudomonas* phage vB_PaeP_Tr60_Ab31, respectively. Both *Puniceispirillum* phage HMO-2011 and *Pseudomonas* phage vB_PaeP_Tr60_Ab31 belong to the family *Podoviridae*. The *terL* phylogenetic tree (Supplementary Fig. S4) showed a distant relatedness to members of the *Podoviridae* clade. Both terminase large and small subunits, together termed the terminase complex, are involved in the cleavage and packaging of concatemeric phage dsDNA^59^. The large terminase subunit is involved in DNA cleavage and translocation into the procapsid while the small terminase subunit is involved in packaging initiation and stimulation of the ATPase activity of the large terminase. These DNA packaging mechanisms are used by most members of the *Caudovirales*.

The translated product of gene 34 was identified as a putative ERF superfamily protein and showed 55% amino acid identity to a homologue encoded by the unclassified *Clostridium* phage phiCP34O (order *Caudovirales*, family *Siphoviridae*). The ERF superfamily proteins are involved in the recombination of phage genomes^60^. The translated product of gene 41 was identified as a putative gp77 and showed 95% amino acid similarity to a homologue encoded by *Mycobacterium* phage Che9d (order *Caudovirales*, family *Siphoviridae*, genus *Che8likevirus*). gp77 proteins are known to function as shut-off genes during early stages of phage replication^61^.

Fifty nine of the translated products of genes in the assembled phage genome showed identity to hypothetical proteins. Of these hypothetical proteins, 56 showed no sequence similarity to known virus families in BLASTp comparison to the RefseqVirus protein database. Three of the genes were predicted to encode glucosaminidase (a hydrolytic enzyme), Phage integrase (a site-specific recombinase that mediates controlled DNA integration and excision) and PDDEXK_1 (nuclease superfamily). Members of this PDDEXK_1 family belong to the PD-(D/E) XK nuclease superfamily. The PD-(D/E)XK nuclease superfamily contains type II restriction endonucleases and many other enzymes involved in DNA recombination and repair^62^.

The protein sequences identified in this analysis indicated the presence of a putative ERF superfamily protein, Phage integrase and PDDEXK_1 family; all proteins implicated in DNA recombination. The ERF superfamily protein encoded by gene 34, whose sequences are expressed during recombination of temperate phages, catalyses annealing of single-stranded DNA chains and pairing of ssDNA with homologous dsDNA, which may function in RecA-dependent and RecA-independent DNA recombination pathways^63^.

A few large contigs contained some predicted ORFs with similarities to phage sequences and coding for specific conserved phage proteins, including terminases, structural proteins (mainly related to Caudovirales tail structures) and phage DNA polymerases (Supplementary Table S2).

### Cluster analysis

Contig datasets from nine metaviromes from various aquatic and soil habitats were selected for dinucleotide frequency comparisons^64^.

**Figure 5:**
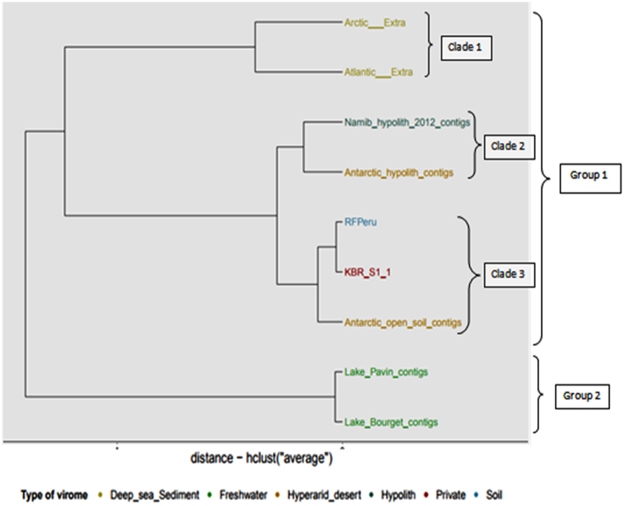
Hierarchical clustering of nine metaviromes (assembled into contigs) based on dinucleotide frequencies. The types of biome are differentiated by colour with Kogelberg Biosphere Reserve - red, freshwater - dark green, hyperarid desert - light blue, hypersaline - yellow, hypolith - dark blue, seawater - light green and unknown biomes - gold. The x-axis denotes eigenvalues distances. The tree was constructed using MetaVir server pipeline according to the method in^64^. More details on sample names are described in supplementary Table S3.

A comparison of the dinucleotide frequencies of the 9 metaviromes shows a clear bimodal clustering (Fig. 5). Group 1, composed of soil-associated habitat and deep sea sediment metaviromes, is further subdivided into soil, hypolith and sediments clades. Group 2 was restricted to freshwater habitats. The Arctic and Atlantic deep sea sediment and freshwater lake^28^ metaviromes clustered in single independent nodes. Such clustering reflects significant genetic similarity between these metaviromes, despite the geographical distances between sample locations.

Both hypolithic metaviromes (i.e., cold Antarctic and hot Namib Desert hypolithic biomass samples) clustered as a single node, despite their widely differing habitat-associated environmental characteristics (dominated by an est. 50 °C mean annual temperature difference) and substantial spatial separation (approx. 55 degrees of latitude), suggesting that aridity and not temperature may be the dominant driver of host and viral diversity^22,65^. Interestingly, soil related metaviromes (from Kogelberg Biosphere Reserve fynbos soil, Peruvian rainforest soil and Antarctic Dry Valley desert soil) clustered together and were clearly distinct from soils which were geographically much closer.

The Kogelberg Biosphere Reserve soil metavirome clustered at a single sub-node with the Peruvian rainforest soil metavirome. Both of these habitats experience high annual rainfall and warm temperatures and are characterised by heavily leached and low nutrient status soils, suggesting that soil composition and/or nutrient status may be the strong driver of the host and viral diversity^66,67^. These observations suggest a niche-dependent pattern, where spatially distinct niche environments cluster together and separate from their geographically closer soil counterparts^65^.

Previous study reported that cluster analysis of hypolith and open soil metaviromes from Antarctic and Namib Desert soil has shown that both hypolith metaviromes clustered at a single node and also that both open soil metaviromes displayed an identical pattern^65^. Similarly to our study, related habitat types harboured more closely related viral communities, despite the great geographic distances or differing environmental conditions. The common factor in these hyperarid environments is water scarcity, which may be a key driver of community speciation and recruitment in these environments. We conclude that these adaptations and the nature of soil habitat compared to the ‘refuge’ habitat of quartz stones for hypolithic communities, may be the driving force between both communities not to cluster together.

### Functional Properties of The Kogelberg Biosphere Reserve Fynbos Soil Metavirome

**Figure 6:**
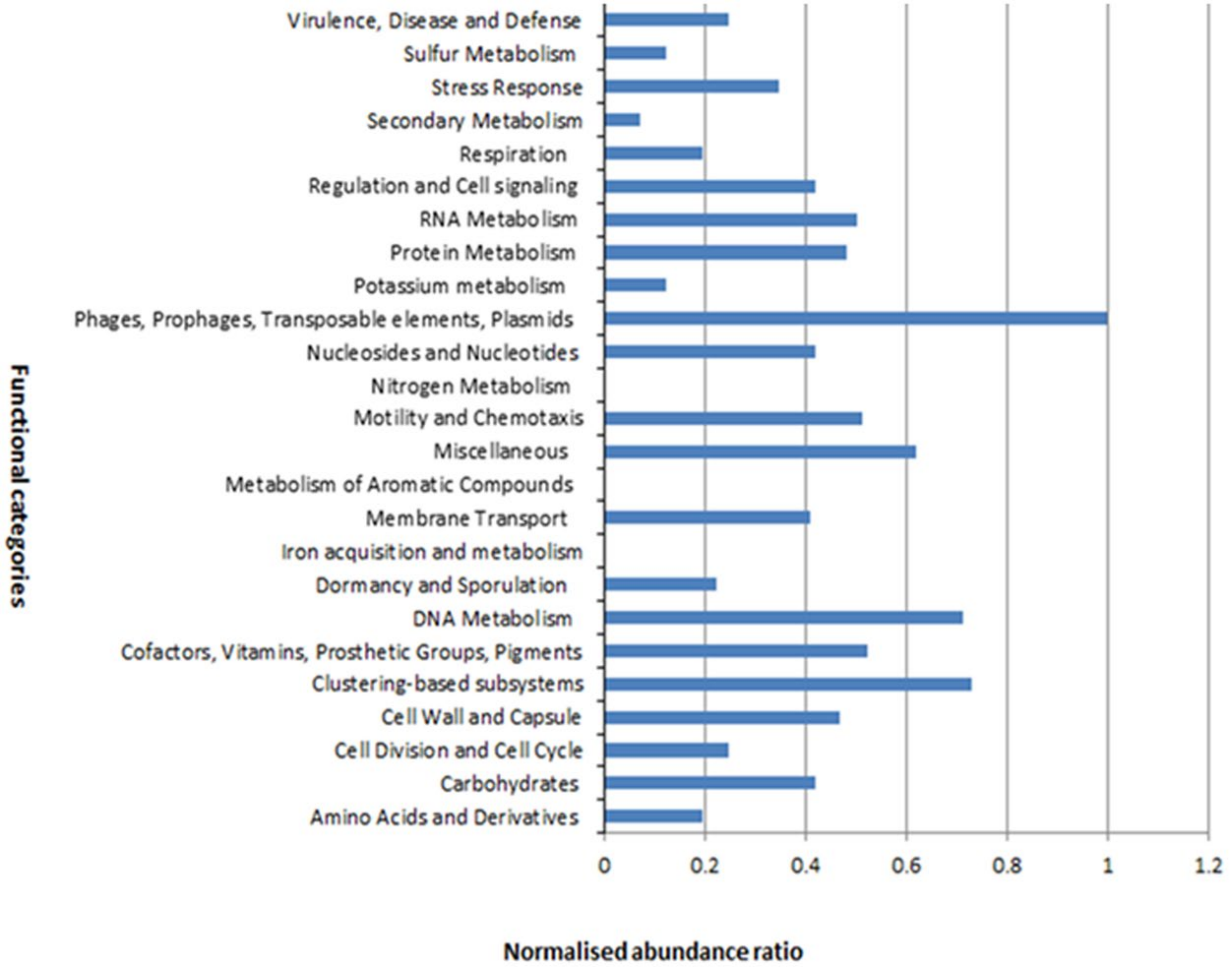
Functional assignment of predicted ORFs. Functional annotation was performed at 60% similarity cut-off as predicted by MG-RAST.

The functional implication of the reads was explored using MG-RAST. The Kogelberg Biosphere Reserve metavirome sequences exhibited a high proportion of uncharacterized ORFs, with 2,362,076 sequences showing no significant similarities to proteins in the databases (ORFans). Twelve functional categories were annotated by MG-RAST, each subdivided into distinct subsystems (Fig. 6). The database searches against SEED in the MG-RAST subsystem resulted in 9360 hits. The highest percentage hits (20.3%) in the functional annotation belonged to the “Phage, prophages, transposable elements and plasmids” subsystem category, with r1t-like streptococcal phages, phage packaging machinery and phage replication annotations most commonly identified.

The other functional subsystem categories showed “Clustering-based subsystems (e.g., biosynthesis of galactoglycans and related lipopolysaccharides; catabolism of an unclassified compound etc., and other clusters identified as unclassified). The “Protein metabolism” and “DNA metabolism” functional categories were also dominant annotations. Many proteins in these functional categories, such as terminases, HNH homing endonucleases, DNA helicases, DNA polymerases and DNA primases, could potentially be of phage origin. These functional groups have also been found to be highly represented in previous metaviromic datasets^23,68,69^.

Analysis of the metavirome reads using the KEGG Orthology (KO) database showed metabolism protein families (carbohydrate metabolism, amino acid metabolism and nucleotide metabolism) to be the most commonly identified. Members of the genetic information procession protein family, including replication and repair, transcription and translation proteins, were also commonly identified. Deeper analysis of a subset of annotated contigs identified genes encoding numerous virus structures (e.g., phage capsid, terminase, tail fibre protein etc.) and DNA manipulating enzymes (e.g., endonuclease, DNA methylase, primase-polymerase, DNA primase/helicase, DNA polymerase I, integrase, ssDNA annealing protein, exonuclease, transferase, site-specific DNA methylase, ligase, recombinase etc.).

From this analysis, we demonstrate that phage-related genes and metabolic genes are highly represented. The virome displayed a strong enrichment in phage-like genes (e.g. phages, prophages, transposable elements, plasmids) and lacked typical cellular categories rarely observed in sequenced phages (e.g. ‘cofactors, vitamins, prosthetic groups, pigments’). Cellular categories commonly identified in known phages were retrieved (e.g. ‘nucleosides and nucleotides’, ‘DNA metabolism’). The highly abundance of virome-associated metabolic genes shows that the phages may have the potential to interfere with the metabolism of their hosts. Our virome analysis, consistent with other virome studies, demonstrate the unexpected picture of global ‘viral’ metabolism, suggesting that viruses might actively dictate the metabolism of infected cells on a global scale^69^.

**Figure 7:**
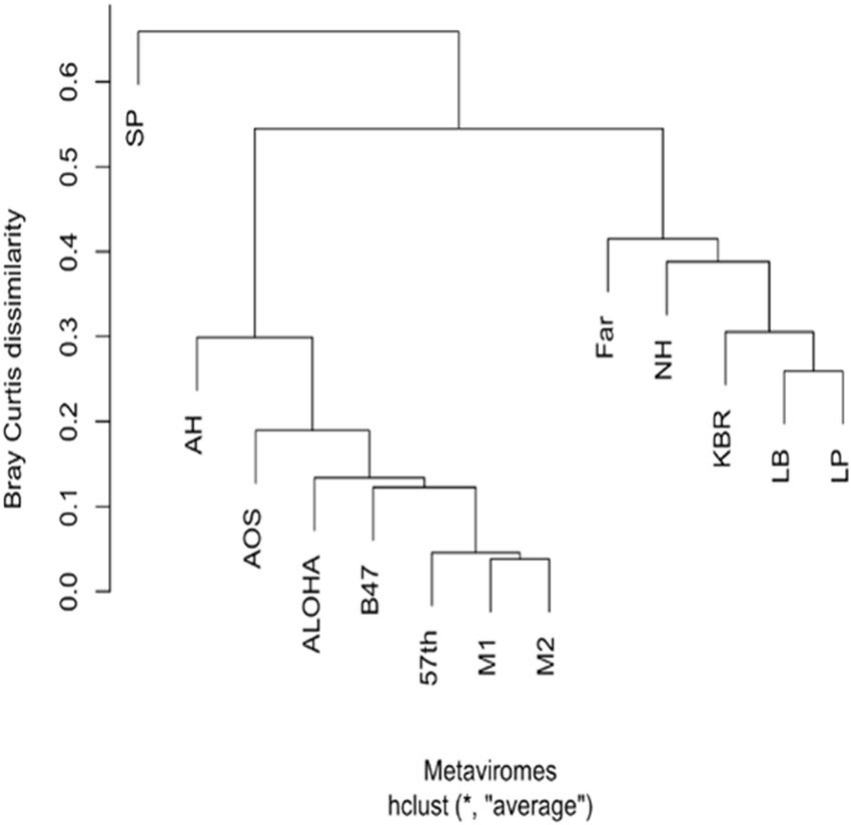
Cluster analysis of functional assignment of predicted ORFs. Viromes were clustered with the hclust algorithm in R according to the abundance of SEED database functional categories present. SEED categories were assigned using Megan6 after blastp-based comparison with the non-redundant protein database of NCBI. More details on the description of metaviromes are described in Supplementary Table 2.

The functional assignments from the SEED database of Kogelberg Biosphere Reserve fynbos soil was clustered with SEED database functional assignments of the 12 previously published metaviromes from both similar and dissimilar environments (fresh water^28^, soil and hypolithic niche communities^22,23^, pond water^27^ and sea water^48^ mentioned in Fig. 2. A cluster analysis of the SEED database subsystem classification revealed different functional patterns between the metaviromes and no clear soil clustering (Fig. 7). The sequences from Kogelberg Biosphere Reserve clustered amongst the sequences from three of the fresh water lakes and the Namib hypolith metaviromes. Antarctic samples (Antarctic open soil and Antarctic hypolith) were more distinct and formed a heterogeneous clade with the other fresh water samples. This can be potentially be explained by the larger number of cellular contamination in some of these metaviromes. This finding suggests that different biomes can share similar functional patterns and, conversely, that taxonomically similar viromes can encode different functional genes. It may also indicate that certain phage groups are more prevalent in certain biogeographic regions.

This study is not without limitations. The major limitation to this study is the use of only a single virome that includes only double stranded DNA viruses.

## Conclusion

We have successfully used the metaviromics approach to explore the diversity and functional composition of a previously unexplored Kogelberg Biosphere Reserve fynbos soil virome. Our quantitative comparison of taxonomic and functional composition of the Kogelberg soil metavirome with other published viromes is a valuable and novel contribution that will enhance the repertoire of publicly available datasets and advance our understanding of viral ecology. Furthermore, contigs corresponding to novel virus genomes were assembled in the current work; this presents an opportunity for future studies aimed at targeting these novel genetic resources for applied biotechnology.

## Experimental Design

### Sample Site Location

Samples were collected from the Kogelberg Biosphere Reserve, situated to the east of Cape Town, South Africa in the Boland Mountains (GPS coordinates: 34°19′48′′.0 S, 18°57′21.0′′ E). Open soil samples were collected aseptically during the winter of 2014. Approximately 20 kg of soil was collected at depth of 0–4 cm. Soil samples were stored in sterile containers at −80 °C.

### Sample Processing, Dna Extraction

Samples were collected in the open soil. Only 3 samples were collected. The DNA of these samples where pooled together for NGS sequencing. Soil samples were processed as previously described^70^ with some modifications. 8 kg of soil and 1X SM buffer (8 L) (0.1 M NaCl, 8 mM MgSO_4_, 50 mM Tris-HCl, pH 7.5) were mixed and shaken vigorously in a sterile container until soil was well suspended and left overnight at 4 °C to settle. The supernatant was centrifuged at 10000 g for 15 min to pellet any remaining soil particles and other debris and passed through a 0.22 µm filter (Millipore, streicup 500 ml). The filtrate was treated with DNase. Viral particles were precipitated with 10% (w/v) polyethylene glycol (PEG) 8000 overnight at 4 °C and centrifuged for 15 min at 11000 g. After removing the supernatant the viral pellet was resuspended in TE buffer, pH 7.6^70^.

The absence of bacterial and eukaryotic DNA was confirmed by PCR with primers pairs E9F (5′-GAG TTT GAT CCT GGC TCA G-3′) and U1510R (5′-GGT TAC CTT GTT ACG ACT T-3′) and ITS1 (5′- TCCGTAGGT GAACCTGCGG-3′) and ITS4 (5′- TCCTCCGCTTATTGATATGC-3′)^71^.

### Transmission Electron Microscopy

Aliquots of viral suspensions isolated from soil were fixed with 2% glutaraldehyde for three hours at 4 °C and 10 µl of the phage suspension was overlaid on a carbon coated grid of 200 Mesh^72^. The suspension was allowed to dry on the grid, which was then negatively stained with 2% uranyl acetate. Excess stain was removed using filter paper and allowed to air-dry prior to examination using a Philips (FEI) CM100 TEM.

### Dna Extraction Sequencing

DNA was extracted from virus particle preparations using a ZR soil microbe DNA MidiPrep^TM^ kit according to manufacturer’s instructions (Zymo Research). Extracted metaviromic DNA (unamplified) was sequenced using an Illumina MiSeq platform (Inqaba Biotechnical Industries). Briefly, following DNA quantification using NanoDrop Fluorospectrometer 3300, 1 ng of isolated metavirome DNA was used to prepare 4 individually indexed NexteraXT libraries. They were then sequenced using the MiSeq v3 (600 cycles) sequencing kit, generating 2 × 300 bp reads. The raw reads were trimmed and demultiplexed, resulting in four fastq files.

### Sequence Data Analysis

The quality of the raw read files was checked with CLC Genomics Workbench version 6.0.1 (CLC, Denmark). The reds were then filtered and trimmed, with the removal of low quality (sequence limit of 0.05), ambiguous reads (maximal of 2 and minimum length of 15). This yielded 1,488,462,918 reads with an average length of 212.05 bp. The post-QC reads were assembled using CLC Genomics Workbench as paired files (3 × 2 read files per site). The assembly resulted in 28,511,204 contigs with a minimum length of 1,002 bases at an N50 of 2,047 and a maximum of 47,854 bases.

The processed reads were assembled *de novo* using CLC Genomics Workbench version 6.0.1 using the default settings. Reads and contigs were uploaded to the MetaVir^37^ (http://metavir-meb.univ-bpclermont.fr), VIROME (http://virome.dbi.udel.edu/)^38^ and MG-RAST (http://metagenomics.anl.gov/)^39^ servers for virus diversity estimations. The viromes were uploaded in 2015 and analysed in 2017. The taxonomic composition was computed from a BLAST comparison with the Refseq complete viral genomes protein sequence database from NCBI (release of 2016-01) using BLASTp with a threshold of 50 on the BLAST bitscore. The assembled sequences were searched for open reading frames (ORFs) and compared to the RefSeq complete viral database using MetaVir and MG-RAST. Functional and organism assignments were based on annotation and other information obtained from the following databases: GenBank, Integrated Microbial Genomes (IMG), Kyoto Encyclopaedia of Genes and Genomes (KEGG), Pathosystems Resource Integration Center (PATRIC), RefSeq, SEED, Swiss-Prot, tremble, and eggnog; and for the assignment of functional hierarchy, COG (clusters of orthologous groups), KEGG Orthology (KO), and NOG databases were used. The Genome relative Abundance and Average Size (GAAS)^73^ tools were used for normalization of the total composition, estimation of the mean genome length and for the estimation of relative abundance and size for each taxon. The phylogenetic tree were generated by an open-source JavaScript library called jsPhyloSVG^74^. The phylogenetic trees were based on the reference sequences and the Kogelberg Biosphere Reserve virome sequences, and computed with 100 bootstraps. Further analysis of the sequences was performed using METAGENassist (a web server that provides a broad range of statistical tools for comparative metagenomics)^75^. Functional assignments produced by VIROME using 120 identified functional subsystems were used for the statistical analysis with METAGENassist.

Clustering analysis comparison was plotted as a clustering tree and computed with pvclust computed by MetaVir (an R package for assessing the uncertainty in hierarchical clustering)^76^ (Fig. 6). Hierarchical clustering using dinucleotide comparisons was used to quantify the grouping behaviour of nine published metaviromes and the comparison were plotted and demonstrated as a clustering dendrograms. Only metaviromes containing more than 50,000 sequences and with an average sequence length of over 100 bp were used, as this comparison is based on a normalised virome sub-sample. Metaviromes that did not match these criteria were not listed for nucleotide composition bias comparison. Hence, only 9 metaviromes were suitable for comparison using dinucleotide frequencies in the MetaVir sever. The largest contigs were analysed by MetaVir. The SEED classification clustering of the 12 metaviromes was assessed using BLASTp against the nr database of NCBI (release 2017-05)^77^. Differences between the virome SEED functional components were transformed into a Bray Curtis dissimilarity matrix using the vegan package in RStudio, clustered using the hclust algorithm (method = average), and represented as a dendrogram^78,79^.

### Data availability

Viral sequences from Kogelberg Biosphere Reserve fynbos soil sample are available on MetaVir under the project KBR under the names “KBR 1 and KBR 2”.

## Electronic supplementary material

Supplementary Information
