# Supplementary material for: Exploring Viral Diversity in a Unique South African Soil Habitat
Source: Sci Rep. 2018 Jan 8;8:111. doi: 10.1038/s41598-017-18461-0 (PMC5758573; doi:10.1038/s41598-017-18461-0)
Supplement: Supplementary file 1 — Supplementary Information [file 41598_2017_18461_MOESM1_ESM.pdf]

SUPPLEMENTARY PAPER

Exploring Viral Diversity In A Unique South African Soil Habitat

Jane Segobola[1,2], Evelien Adriaenssens[2], Tsepo Tsekoa[1], Konanani Rashamuse[1] and Don Cowan[2]

[1]Biosciences Unit, Council for Scientific and Industrial Research (CSIR), Pretoria, South Africa

[2] Centre For Microbial Ecology and Genomics, University Of Pretoria, Pretoria, South Africa

Corresponding Author: : Don Cowan, Centre for Microbial Ecology and Genomics, Department of Genetics, University of Pretoria, Hatfield 0028, Pretoria, South Africa. Tel: +27 (12) 420 5873, don.cowan@up.ac.za

SUPPLEMENTARY FIGURES:

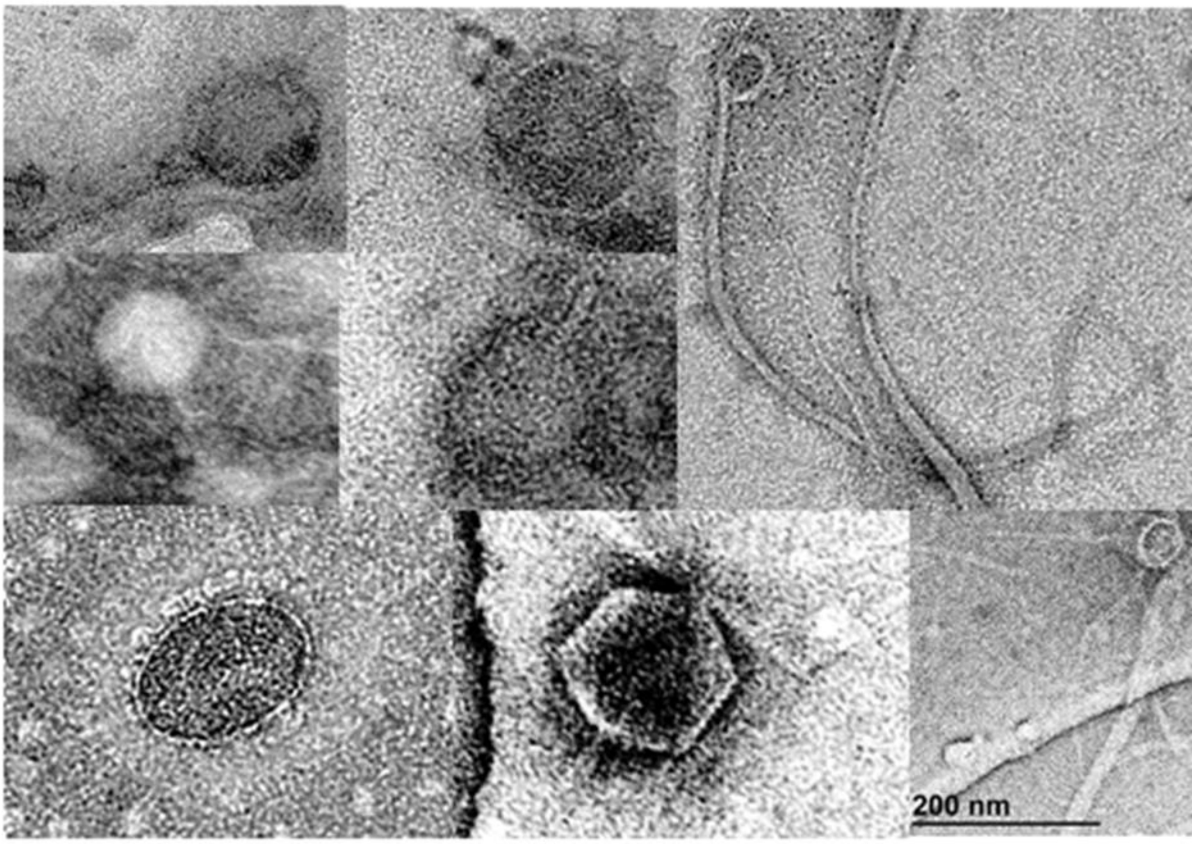

Supplementary Figure S1: Transmission electron micrographs of the viral particles obtained from KBR. Scale bars correspond to 200 nm. Particles were negatively stained with 2% uranyl acetate. Virus particles observed either belong to the families *Siphoviridae, Myoviridae* or are of an undetermined shape (VLP)

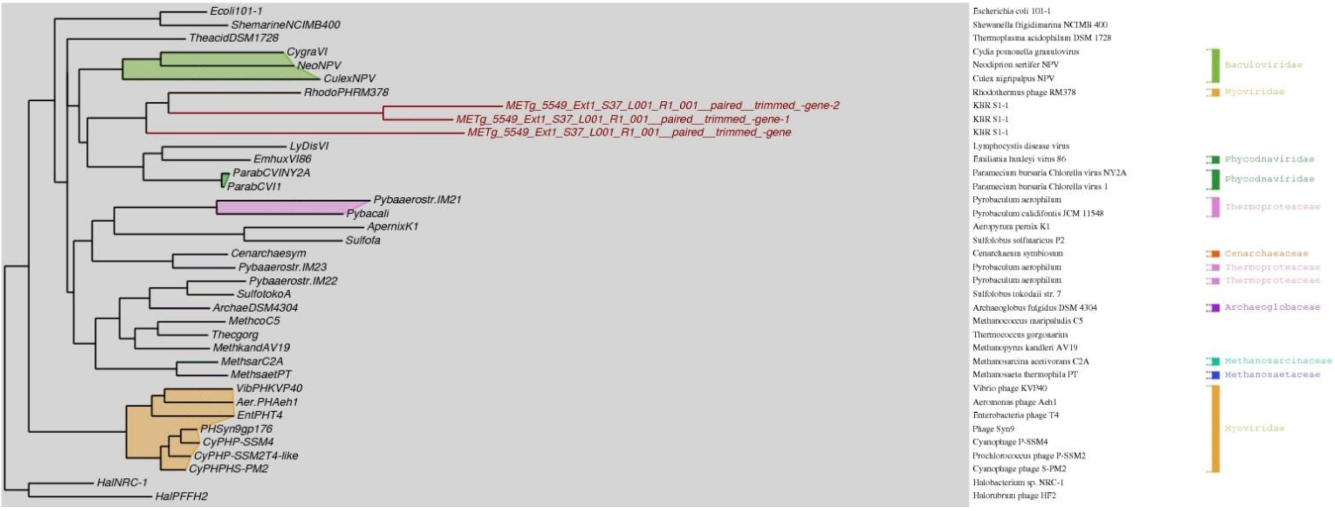

Supplementary Figure S2: Phylogenetic tree of Pol B Marker gene amino acid sequences of the Kogelberg Biosphere reserve sequences – Red. Reference sequences are coloured according to their taxonomy: *Baculoviridae* – light green, *Myoviridae* – yellow, *Phycodnaviridae* – Dark green, *Thermoproteaceae* – Pink, *Cenarcheaceae* – Mustard, *Archaeoglobaceae* – Purple, *Methanosarcinaceae* – Turquoise, and *Methanosaetaceae* – Blue. Sequences were aligned with de novo assembly using CLC genomics workbench version 6.0.1 (CLC, Denmark) and visualised with MetaVir server [1]. Each tree is computed with 100 bootstraps, and the resulting values are indicated for each node. Scale bar indicates the number of substitutions per site.

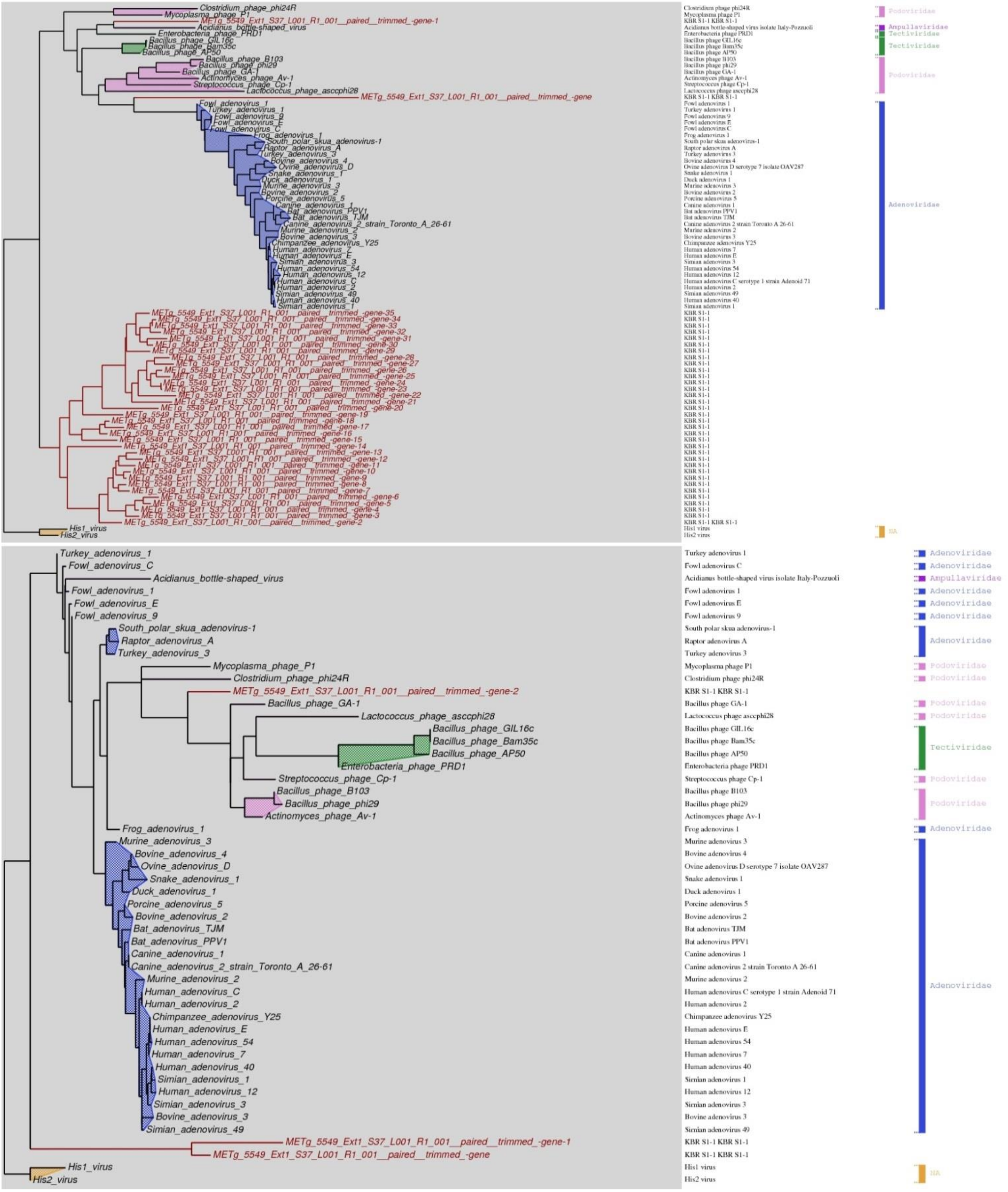

Supplementary Figure S3: Phylogenetic tree of Pol B1 Marker gene amino acid sequences of the Kogelberg Biosphere reserve sequences – Red. Reference sequences are coloured according to their taxonomy: *Podoviridae* – Pink, *Ampullariidae* – Purple, *Tectiviridae* – green, *Adenoviridae* – Blue, Not assigned any Family – Yellow. Sequences

were aligned with de novo assembly using CLC genomics workbench version 6.0.1 (CLC, Denmark) and visualised with MetaVir server [1]. Each tree is computed with 100 bootstraps, and the resulting values are indicated for each node. Scale bar indicates the number of substitutions per site.

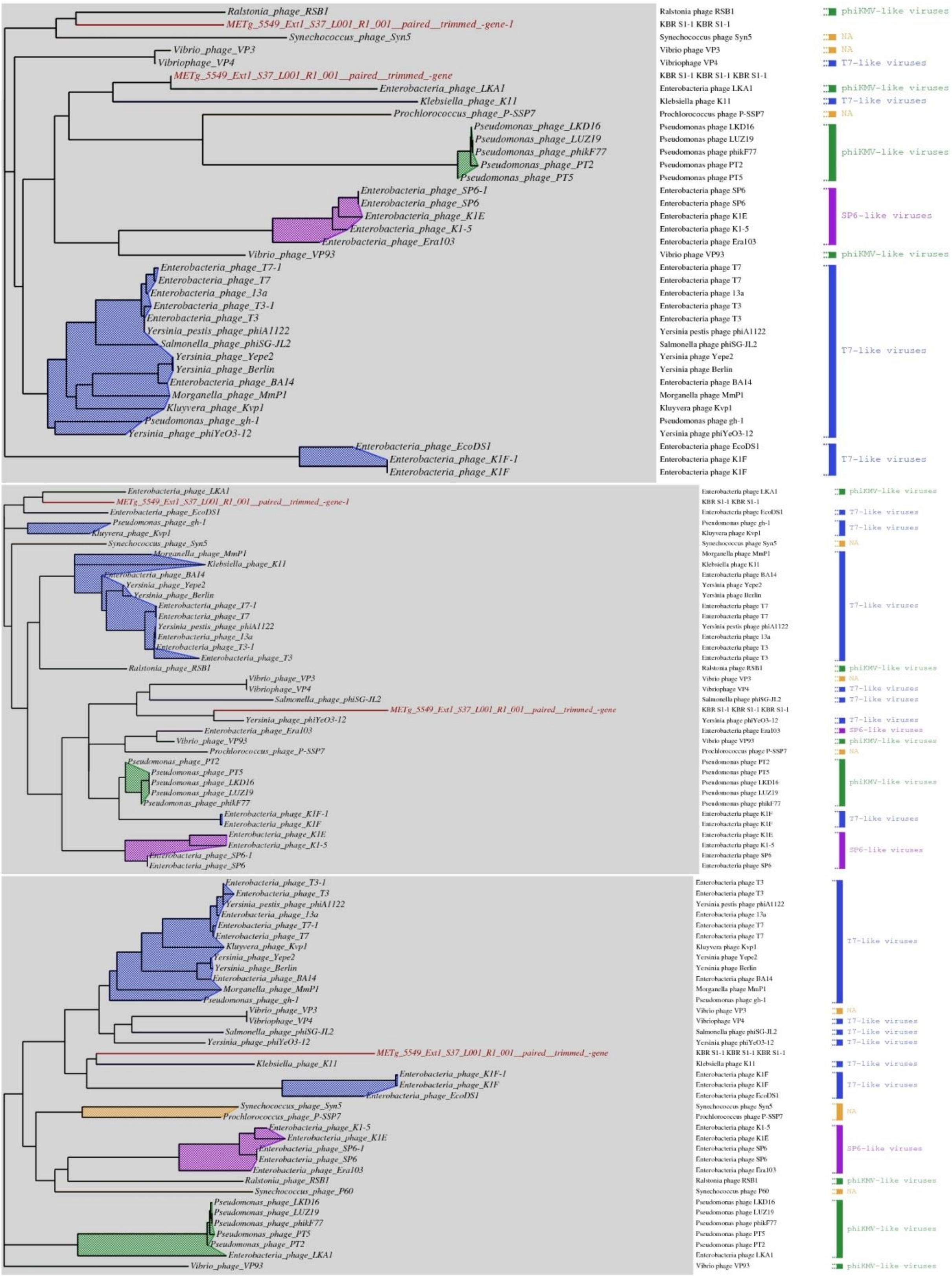

Supplementary Figure S4: Phylogenetic tree of T7gp17 Marker gene amino acid sequences of the Kogelberg Biosphere reserve sequences – Red. Reference sequences are coloured according to their taxonomy: phiKMV-like viruses – green, T7-like viruses – Blue,

SP6-like viruses – Purple, not assigned any family. Sequences were aligned with de novo assembly using CLC genomics workbench version 6.0.1 (CLC, Denmark) and visualised with MetaVir server [1]. Each tree is computed with 100 bootstraps, and the resulting values are indicated for each node. Scale bar indicates the number of substitutions per site.

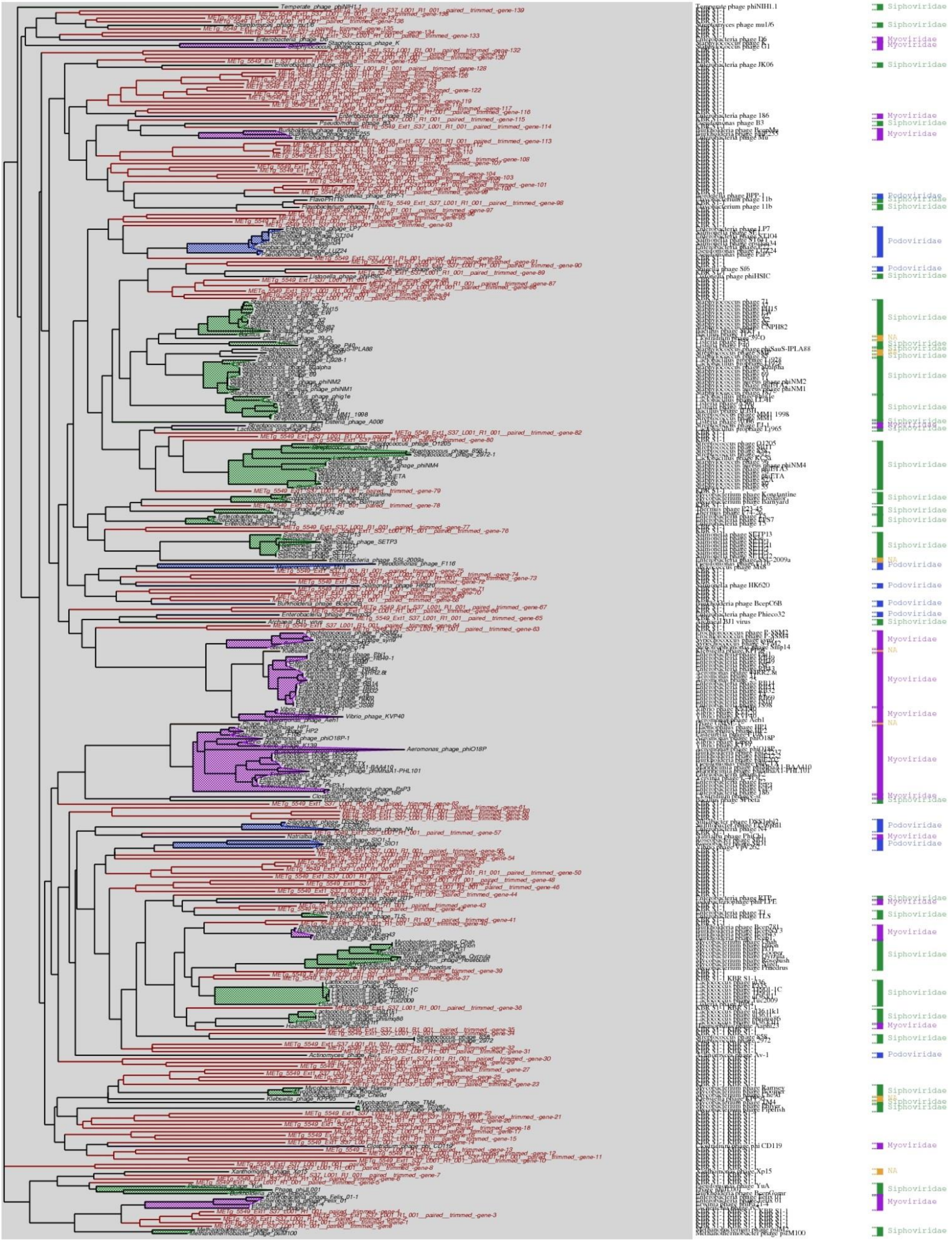

Temperate phage phiNIH1.1
Siphoviridae
Myoviridae
Podoviridae
NA

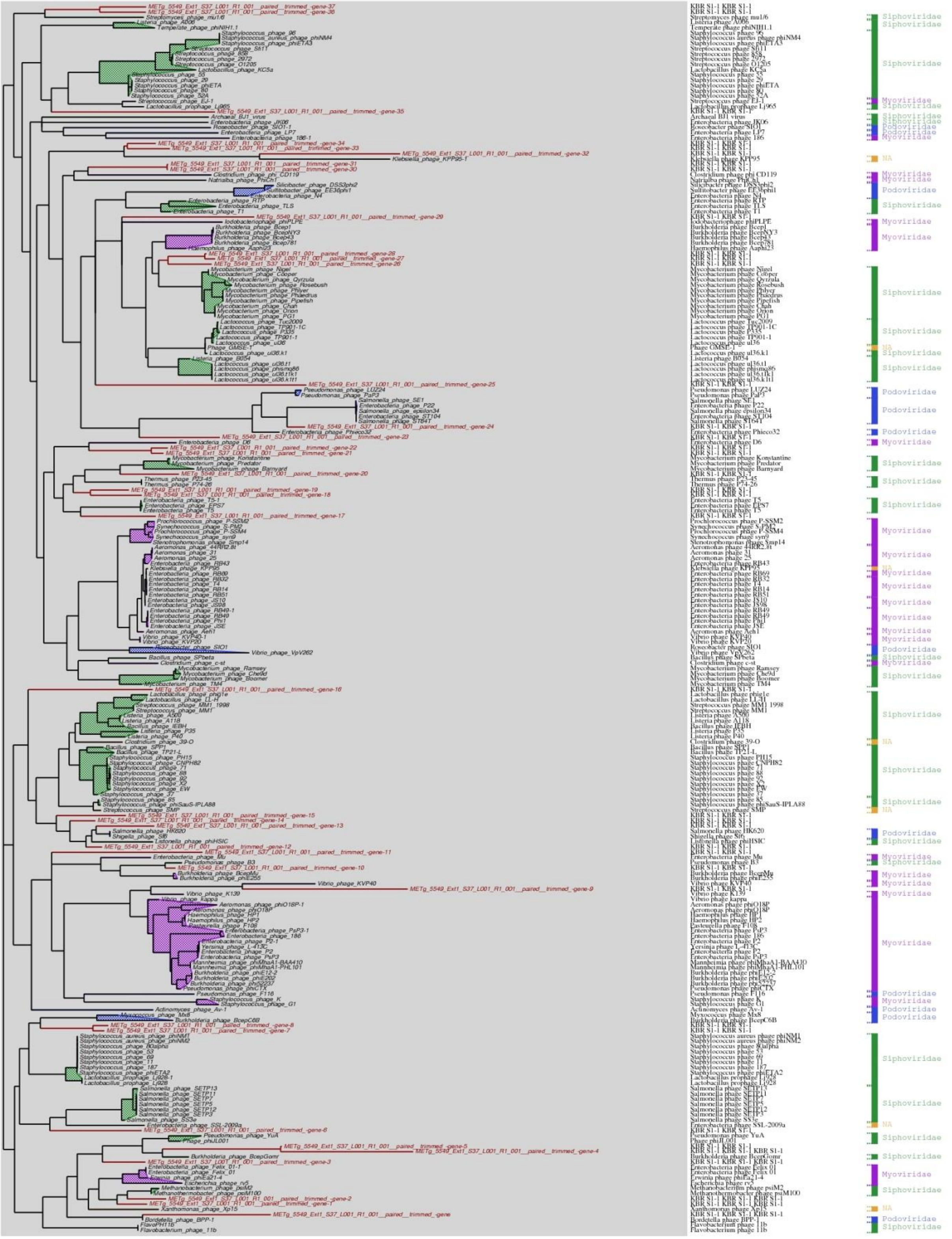

Supplementary Figure S5: Phylogenetic tree of TerL Marker gene amino acid sequences of the Kogelberg Biosphere reserve sequences – Red. Reference sequences are coloured according to their taxonomy: *Siphoviridae* – Green, *Myoviridae* – Purple, *Podoviridae* – Blue, Not assigned family – yellow Sequences were aligned with de novo

assembly using CLC genomics workbench version 6.0.1 (CLC, Denmark) and visualised with MetaVir server [1]. Each tree is computed with 100 bootstraps, and the resulting values are indicated for each node. Scale bar indicates the number of substitutions per site.

Supplementary Table S1: Comparisons of functional and taxonomic analysis between VIROME and MetaVir

**Functional analysis**	**VIROME**	**MetaVir**
Hypothetical proteins	1359	46457
glycoside hydrolase	124	83
gp11	11	39
YapH protein	1	2
HNH homing endonuclease	6	26
Helicase	231	277
Primase	77	93
pyrophosphatase	2	1
DNA polymerase	417	287
terminase large subunit	95	316
Lysine	19	95
endonuclease	117	198
structural protein	61	101
phage-related protein	52	7
**Taxonomic analysis**	**VIROME**	**MetaVir**
Caudovirales	1118	25922

Phycodnaviridae	20	499
Ampullaviridae	1	173
Mimiviridae	1	110

Supplementary Table S2: Sample description of taxonomic abundance of the 10 largest contigs

Contigs name	Contigs length	#of predicted genes	Predicted genes	#Hypothetical proteins
5549_Ext1_S37_L001_R1_001__paired__trimmed__p_414	47854	63	terminase, glucosaminidase, phage_integrase	57
5549_Ext1_S37_L001_R1_001__paired__trimmed__p_53	44760	58	putative terminase large subunit, Peptidase_S74, endolysin, Lipase, putative DNA polymerase, putative DNA helicase putative adenine methyltransferase	48
5549_Ext1_S37_L001_R1_001__paired__trimmed__p_458	42564	61	putative endonuclease, putative terminase large subunit	52
5549_Ext1_S37_L001_R1_001__paired__trimmed__p_185	42057	53	putative phage terminase large subunit, putative peptidase, putative lytic tail protein, putative endolysin, putative DNA methyltransferase, putative DNA helicase, putative RecB family exonuclease, putative primase, putative DNA polymerase	27

5549_Ext1_S37_L001_R1_001__paired__trimmed__p_9	41177	68	putative endolysin, putative terminase large subunit	53
5549_Ext1_S37_L001_R1_001__paired__trimmed__p_90	40336	49	putative terminase large subunit, putative endoprotease, putative major capsid protein, putative endolysin, putative DNA polymerase	33
5549_Ext1_S37_L001_R1_001__paired__trimmed__p_259	36396	45	Pectate_lyase, Endonuclease, putative terminase large subunit	39
5549_Ext1_S37_L001_R1_001__paired__trimmed__p_645	36310	87	RNA_ligase, Endonuclease, putative deoxycytidylate deaminase, putative PseT polynucleotide 5'-kinase and 3'-phosphatase, putative RNA ligase	67
5549_Ext1_S37_L001_R1_001__paired__trimmed__p_929	34830	43	putative phage tail fiber-like protein, putative tail fiber protein, putative N-acetylmuramoyl-L-alanine amidase, putative DNA polymerase B region, putative tRNA ribotransferase, putative GTP cyclohydrolase, putative glutamine amidotransferases class-II, putative organic radical activating enzyme	21
5549_Ext1_S37_L001_R1_001__paired__trimmed__p_416	30995	34	putative Mu-like prophage I protein, putative tape measure protein, Pectate_lyase, putative DNA-binding protein	24

Supplementary Table S3: Selected Biomes for viral composition comparison with KBR

Table discription	Sample name	Project ID	Sample type	References
**KBR (s)**	KBR sample 1	5549	soil	Current study
**AOS (s)**	Antarctic open soil contigs	2473	soil	[2]
**RF(s)**	RF Peru	4906	Soil	[3]
**AH**	Antarctic hypolith contigs	2472	hypolith	[2]
**NH**	Namib hypolith contigs	2186	hypolith	[4]
**Far (fw)**	Far-T4 Lake Pavin	5127	fresh water	[5]
**LB (fw)**	Lake Bourget	1327	fresh water	[6]
**LP (fw)**	Lake Pavin	1328	fresh water	[6]
**57th (fw)**	57th_St_05-Jun-13	3305	fresh water	[7]
**M1 (fw)**	Montrose_05-Jun-13-1	3306	fresh water	[7]
**M2 (fw)**	Montrose_25-Jun-13-2	3307	fresh water	[7]
**SP (p)**	Salted pond	25	pond	[8]
**ALOHA (ds)**	ALOHA_station_deep_abyss	3816	deep sea	[9]
**B47 (ds)**	B47_Bohai_Sea_Sep_2010	5754	sea	[9]
**P**	P._acuta_2012	2315	unknown	[10]
**Sup05**	Sup05_prophage_contigs	3232	unknown	[11]
**VS**	VirSorter_curated_dataset	5062	unknown	[12]
**10eld**	10_eld_contigs		unknown	[13]

REFERENCES

1. Roux, S., Tournayre, J., Mahul, A., Debroas, D. & Enault, F. Metavir 2: new tools for viral metagenome comparison and assembled virome analysis. *BMC Bioinformatics* **15,** 76 (2014).

2. Zablocki, O., Adriaenssens, E. & Cowan, D. Diversity and ecology of viruses in hyperarid desert soils. *Appl. Environ. Microbiol.* **82,** AEM.02651-15- (2015).

3. Fierer, N. *et al.* Metagenomic and small-subunit rRNA analyses reveal the genetic diversity of bacteria, archaea, fungi, and viruses in soil. *Appl. Environ. Microbiol.* **73,** 7059–7066 (2007).

4. Adriaenssens, E. M. *et al.* Metagenomic analysis of the viral community in Namib Desert hypoliths. *Environ. Microbiol.* **17,** 480–495 (2015).

5. Roux, S. *et al.* Analysis of metagenomic data reveals common features of halophilic viral communities across continents. *Environ. Microbiol.* **18,** 889–903 (2015).

6. Roux, S. *et al.* Assessing the Diversity and Specificity of Two Freshwater Viral Communities through Metagenomics. *PLoS One* **7,** e33641 (2012).

7. Watkins, S. C. *et al.* Assessment of a metaviromic dataset generated from nearshore Lake Michigan. *Mar. Freshw. Res.* **67,** 1700 (2016).

8. Rodriguez-Brito, B. *et al.* Viral and microbial community dynamics in four aquatic environments. *Isme J* **4,** 739–751 (2010).

9. Angly, F. E. *et al.* The marine viromes of four oceanic regions. *PLoS Biol.* **4,** e368 (2006).

10. Wood-Charlson, E. M., Weynberg, K. D., Suttle, C. A., Roux, S. & van Oppen, M. J. H. Metagenomic characterization of viral communities in corals: mining biological signal from methodological noise. *Environ. Microbiol.* **17,** 3440–9 (2015).

11. Roux, S. *et al.* Ecology and evolution of viruses infecting uncultivated SUP05 bacteria as revealed by single-cell- and meta-genomics. *Elife* **3,** 1–20 (2014).

12. Roux, S., Hallam, S. J., Woyke, T. & Sullivan, M. B. Viral dark matter and virus-host interactions resolved from publicly available microbial genomes. *Elife* **4,** e08490 (2015).

13. Kim, M.-S., Park, E.-J., Roh, S. W. & Bae, J.-W. Diversity and Abundance of Single-Stranded DNA Viruses in Human Feces. *Appl. Environ. Microbiol.* **77,** 8062–8070 (2011).
